# Health literacy and tuberculosis control: systematic review and meta-analysis

**DOI:** 10.2471/BLT.23.290396

**Published:** 2024-03-27

**Authors:** Arohi Chauhan, Malik Parmar, Girish C Dash, Sandeep Chauhan, Krushna C Sahoo, Kajal Samantaray, Jessica Sharma, Pranab Mahapatra, Sanghamitra Pati

**Affiliations:** a Public Health Foundation of India, New Delhi, India.; b Country Office for India, World Health Organization, New Delhi, India.; c Indian Council of Medical Research, Regional Medical Research Centre, Chandrasekharpur, Bhubaneswar, Odisha 751023, India.; d WHO National Tuberculosis Elimination Programme Technical Support Network, New Delhi, India.; e Department of Psychiatry, Kalinga Institute of Medical Sciences, Bhubaneswar, India.

## Abstract

**Objective:**

To identify literature on health literacy levels and examine its association with tuberculosis treatment adherence and treatment outcomes.

**Methods:**

Two authors independently searched Pubmed®, Embase, CINAHL, PsycINFO, Scopus, LILACS, Global Health Medicus and ScienceDirect for articles reporting on health literacy levels and tuberculosis that were published between January 2000 and September 2023. We defined limited health literacy as a person's inability to understand, process, and make decisions from information obtained concerning their own health. Methodological quality and the risk of bias was assessed using the JBI critical appraisal tools. We used a random effects model to assess the pooled proportion of limited health literacy, the association between health literacy and treatment adherence, and the relationship between health literacy and tuberculosis-related knowledge.

**Findings:**

Among 5813 records reviewed, 22 studies met the inclusion criteria. The meta-analysis revealed that 51.2% (95% confidence interval, CI: 48.0–54.3) of tuberculosis patients exhibit limited health literacy. Based on four studies, patients with lower health literacy levels were less likely to adhere to tuberculosis treatment regimens (pooled odds ratio: 1.95; 95% CI: 1.37–2.78). Three studies showed a significant relationship between low health literacy and inadequate knowledge about tuberculosis (pooled correlation coefficient: 0.79; 95% CI: 0.32–0.94).

**Conclusion:**

Health literacy is associated with tuberculosis treatment adherence and care quality. Lower health literacy might hamper patients' ability to follow treatment protocols. Improving health literacy is crucial for enhancing treatment outcomes and is a key strategy in the fight against tuberculosis.

## Résumé

**Objectif:**

Identifier la littérature consacrée aux niveaux d'éducation à la santé et étudier ses liens avec l'adhésion au traitement de la tuberculose ainsi qu'avec l'issue de ce traitement.

**Méthodes:**

Deux auteurs ont exploré Pubmed®, Embase, CINAHL, PsycINFO, Scopus, LILACS, Global Health Medicus et ScienceDirect, indépendamment l'un de l'autre, à la recherche d'articles portant sur les niveaux d'éducation à la santé et la tuberculose, publiés entre janvier 2000 et septembre 2023. Nous avons défini qu'un faible niveau d'éducation à la santé signifiait qu'une personne était incapable de comprendre, d'assimiler et de prendre des décisions sur la base des informations qui lui étaient communiquées concernant sa santé. Nous avons utilisé les outils d'évaluation critique du JBI pour déterminer la qualité méthodologique et le risque de biais. Enfin, nous avons appliqué un modèle à effets aléatoires pour calculer la proportion groupée correspondant à un faible niveau d'éducation à la santé, le rapport entre l'éducation à la santé et l'adhésion au traitement, et celui entre l'éducation à la santé et les connaissances liées à la tuberculose.

**Résultats:**

Sur 5813 documents examinés, 22 correspondaient aux critères d'inclusion. La méta-analyse a révélé que 51,2% (intervalle de confiance de 95%, IC: 48,0–54,3) des patients tuberculeux affichaient un faible niveau d'éducation à la santé. D'après quatre études, les patients avec un niveau d'éducation à la santé moins élevé avaient moins tendance à suivre les traitements contre la tuberculose (odds ratio groupé: 1,95; IC de 95%: 1,37-2,78). Trois études ont montré un lien significatif entre un faible niveau d'éducation à la santé et des connaissances insuffisantes sur la tuberculose (coefficient de corrélation groupé: 0,79; IC de 95%: 0,32-0,94).

**Conclusion:**

L'éducation à la santé a un impact sur l'adhésion au traitement contre la tuberculose et la qualité des soins. Un niveau d'éducation moindre peut entraver la capacité des patients à suivre les protocoles thérapeutiques. Promouvoir l'éducation à la santé est essentiel pour améliorer les résultats du traitement et constitue un élément clé de la stratégie de lutte contre la tuberculose.

## Resumen

**Objetivo:**

Identificar la bibliografía sobre los niveles de alfabetización sanitaria y examinar su asociación con el cumplimiento terapéutico de la tuberculosis y los resultados del tratamiento.

**Métodos:**

Dos autores buscaron de manera independiente en Pubmed®, Embase, CINAHL, PsycINFO, Scopus, LILACS, Global Health Medicus y ScienceDirect artículos que informaran sobre los niveles de alfabetización sanitaria y de tuberculosis publicados entre enero de 2000 y septiembre de 2023. Se definió la alfabetización sanitaria limitada como la incapacidad de una persona para comprender, procesar y tomar decisiones a partir de la información obtenida en relación con su propia salud. La calidad metodológica y el riesgo de sesgo se evaluaron mediante las herramientas de evaluación crítica de JBI. Se utilizó un modelo de efectos aleatorios para evaluar el porcentaje agrupado de alfabetización sanitaria limitada, la asociación entre la alfabetización sanitaria y el cumplimiento del tratamiento y la relación entre la alfabetización sanitaria y los conocimientos relacionados con la tuberculosis.

**Resultados:**

De los 5813 registros revisados, 22 estudios cumplieron los criterios de inclusión. El metanálisis reveló que el 51,2% (intervalo de confianza del 95%, IC: 48,0-54,3) de los pacientes con tuberculosis presentan conocimientos sanitarios limitados. Según cuatro estudios, los pacientes con niveles más bajos de conocimientos sanitarios tenían menos probabilidades de cumplir los regímenes de tratamiento de la tuberculosis (razón de posibilidades acumulada: 1,95; IC del 95%: 1,37-2,78). Tres estudios mostraron una relación significativa entre los bajos conocimientos sanitarios y los conocimientos insuficientes sobre la tuberculosis (coeficiente de correlación agrupado: 0,79; IC del 95%: 0,32-0,94).

**Conclusión:**

La alfabetización sanitaria está asociada al cumplimiento del tratamiento de la tuberculosis y a la calidad de la atención. Una alfabetización sanitaria baja podría dificultar la capacidad de los pacientes para seguir los protocolos de tratamiento. Mejorar la alfabetización sanitaria es crucial para mejorar los resultados del tratamiento y constituye una estrategia clave en la lucha contra la tuberculosis.

## ملخص

**الغرض:**

التعرف على الأدبيات المتعلقة بمستويات المعرفة الصحية، وفحص ارتباطها بالالتزام بعلاج السل ونتائج العلاج.

**الطريقة:**

قام مؤلفان بالبحث بشكل مستقل في قواعد البيانات Pubmed®‎، وEmbase، وCINAHL، وPsycINFO، وScopus، وLILACS، وGlobal Health Medicus، وScienceDirect، عن مقالات تتحدث عن مستويات المعرفة الصحية والسل، والتي تم نشرها في الفترة ما بين يناير/كانون ثاني 2000، وسبتمبر/أيلول 2023. لقد قمنا بتعريف المعرفة الصحية المحدودة على أنها عدم قدرة الشخص على الاستيعاب، والمعالجة، واتخاذ القرارات بناءً على المعلومات التي تم الحصول عليها فيما يتعلق بصحتهم. تم تقييم الجودة المنهجية وخطر التحيز باستخدام أدوات التقييم النقدي الخاصة بـ JBI. قمنا باستخدام نموذج التأثيرات العشوائية لتقييم النسبة المجمعة للمعرفة الصحية المحدودة، والارتباط بين المعرفة الصحية والالتزام بالعلاج، والعلاقة بين المعرفة الصحية والمعلومات المتعلقة بالسل.

**النتائج:**

من بين 5813 سجلاً تمت مراجعتها، استوفت 22 دراسة معايير الاشتمال. كشف التحليل التلوي أن %51.2 (بفاصل ثقة مقداره %95: 48.0 إلى 54.3) من مرضى السل لديهم معرفة صحية محدودة. استناداً إلى أربع دراسات، فإن المرضى الذين لديهم مستويات منخفضة من المعرفة الصحية كانوا أقل احتمالية للالتزام بنظم علاج السل (نسبة الاحتمالات المجمعة: 1.95؛ ‏بفاصل ثقة مقداره %95: 1.37 إلى 2.78). أظهرت ثلاث دراسات وجود علاقة ملموسة بين المعرفة الصحية المنخفضة وعدم كفاية المعلومات حول مرض السل (معامل الارتباط المجمع: 0.79؛ ‏بفاصل ثقة مقداره %95: 0.32 إلى 0.94).

**الاستنتاج:**

ترتبط المعرفة الصحية بالالتزام بعلاج السل وجودة الرعاية، وذلك لأن المعرفة الصحية المنخفضة قد تعيق قدرة المرضى على اتباع بروتوكولات العلاج. يعد تحسين المعرفة الصحية أمرًا حيويًا لتعزيز نتائج العلاج، وهو استراتيجية رئيسية في سبيل مكافحة السل.

## 摘要

**目的:**

查找健康素养水平相关文献，并研究其与结核病治疗依从性和治疗效果之间的关系。

**方法:**

两位作者各自检索了 Pubmed®、Embase、CINAHL、PsycINFO、Scopus、LILACS、Global Health Medicus 和 ScienceDirect 数据库，以查找 2000 年 1 月至 2023 年 9 月期间发表的关于健康素养水平和结核病的文献资料。我们认为，如果人们无法理解、处理其自身健康相关信息并据此做出决策，则表明其健康素养有限。使用 JBI 的重要评估工具进行了方法学质量和偏倚风险评估。通过使用随机效应模型，我们评估了健康素养有限人群的汇总比例、健康素养与治疗依从性之间的关系以及健康素养与结核病相关知识之间的关系。

**结果:**

在查看 5,813 份记录后，我们发现有 22 项研究符合纳入标准。荟萃分析显示，51.2%（95% 置信区间，CI：48.0–54.3）的结核病患者的健康素养水平比较有限。四项研究的结果显示，健康素养水平较低的患者不太可能遵循结核病治疗方案（合并优势比：1.95；95% 置信区间：1.37-2.78）。三项研究的结果表明，健康素养较低与结核病知识不足之间有明显的关联性（合并相关系数：0.79；95% 置信区间：0.32-0.94）。

**结论:**

健康素养与结核病治疗依从性和护理质量有关，因为健康素养较低可能会导致患者无法遵循治疗方案。提高健康素养对提高治疗效果至关重要，也是在治疗结核病时可采取的一项关键战略。

## Резюме

**Цель:**

Выявление литературы, посвященной уровням медицинской грамотности населения, и изучение ее связи с приверженностью к лечению туберкулеза и результатами лечения.

**Методы:**

Два автора независимо друг от друга изучили базы данных Pubmed®, Embase, CINAHL, PsycINFO, Scopus, LILACS, Global Health Medicus и ScienceDirect с целью поиска статей об уровне медицинской грамотности и туберкулезе, опубликованных в период с января 2000 года по сентябрь 2023 года. Понятие «ограниченная медицинская грамотность» определялось как неспособность человека понимать, обрабатывать и принимать решения на основе полученной информации о своем здоровье. Методологическое качество и риск предвзятости оценивались с помощью инструментов критической оценки JBI. Для оценки совокупной доли лиц с ограниченной медицинской грамотностью, связи между медицинской грамотностью и приверженностью лечению, а также связи между медицинской грамотностью и знаниями о туберкулезе использовалась модель случайных эффектов.

**Результаты:**

Из 5813 просмотренных записей 22 исследования соответствовали критериям включения. Результаты метаанализа показали, что 51,2% (95%-й доверительный интервал, ДИ: 48,0–54,3) пациентов с туберкулезом обладают ограниченной медицинской грамотностью. По данным четырех исследований, пациенты с более низким уровнем медицинской грамотности с меньшей вероятностью будут придерживаться схем лечения туберкулеза (объединенное отношение шансов: 1,95; 95%-й ДИ: 1,37–2,78). Три исследования показали значительную связь между низким уровнем медицинской грамотности и недостаточными знаниями о туберкулезе (суммарный коэффициент корреляции: 0,79; 95%-й ДИ: 0,32–0,94).

**Вывод:**

Медицинская грамотность связана с приверженностью лечению туберкулеза и качеством медицинской помощи, поскольку низкая медицинская грамотность может препятствовать способности пациентов следовать протоколам лечения. Повышение уровня медицинской грамотности имеет решающее значение для улучшения результатов лечения и является одной из ключевых стратегий в борьбе с туберкулезом.

## Introduction

Health literacy is the ability to apply various skills – like reading, counting and problem-solving – to obtain, understand and use health-related information. Such knowledge and skills support informed decision-making, facilitate greater health-care engagement, help navigating health-care systems, reduce health disparities and contribute to lower health-care costs.^1^ While enhanced health literacy is associated with reduced risk behaviours for chronic diseases, improved self-reported health and fewer hospitalizations,^2^ low health literacy is linked to poor treatment adherence, worse health outcomes and increased health-care costs for both individuals and the health system.^3^^,^^4^ Hence, health literacy is a key determinant of a person's health and well-being and has emerged as an important aspect of the successful management and prevention of diseases such as tuberculosis.^5^^,^^6^

Tuberculosis treatment requires daily intake of medication for 4–6 months, and interruption to this schedule can lead to drug resistant tuberculosis, presenting a complex long-term challenge to patients and the health system.^7^ While the directly observed treatment short course therapy has cured millions of tuberculosis patients since the late 1990s,^7^ the impact on lowering tuberculosis incidence and transmission has not been as great as anticipated.^8^ Successfully managing tuberculosis requires clear communication with health-care providers and the cultivation of robust self-care skills;^2^ and misunderstandings about tuberculosis diagnoses, treatment plans and self-care instructions can lead to treatment nonadherence.^6^^,^^9^ Therefore, accessibility and use of health-care information are determinants of a tuberculosis patient’s response to care and subsequent treatment outcomes.^7^^,^^10^ Patients need to access and accurately interpret health-care information; understand referral reasons; implement prevention and care plans; remember drug labels and medication dosages accurately; acknowledge the importance of follow-up appointments and nutrition; and recognize the potential consequences of not adhering to treatment.^9^

Social determinants of health, such as living standards and education, also play a critical role in the onset and worsening of tuberculosis.^10^ Health literacy is one hypothesized mechanism through which level of education affects health outcomes among tuberculosis patients.^11^ Health literacy is a critical component of the social infrastructure (access, use, equity and empowerment) affecting tuberculosis outcomes, and is essential for its prevention, early detection and treatment.

Thus, investment in health literacy is essential to achieve the targets to end tuberculosis. Successful integration of health literacy into tuberculosis policy and services rests on the availability of evidence related to the health literacy. Hence, the aim of this systematic review is to compile information on health literacy levels among individuals with active tuberculosis, examining how health literacy correlates with treatment adherence and outcomes and identifying factors associated with health literacy in the context of active tuberculosis.

## Methods

We performed a systematic review to examine the relationship between health literacy and tuberculosis treatment adherence and outcomes, using the Preferred Reporting Items for Systematic Reviews and Meta-Analysis guidelines. We registered the review with PROSPERO (CRD42023404407). 

### Inclusion criteria

We included primary studies on individuals with active tuberculosis that also reported health literacy levels. Report types such as reviews, editorials, case reports, conference abstracts, theses or unpublished materials were excluded from the analysis. We define tuberculosis as the chronic infectious disease caused by *Mycobacterium tuberculosis*. We evaluated health literacy levels according to the definition: “…individuals’ capacity to obtain, process and understand basic health information and services needed to make appropriate health decisions.”^12^ We included functional, interpretative and critical aspects of health literacy in our review.^12^

### Study selection and review

We searched the electronic databases Pubmed®, Embase, CINAHL, ScienceDirect, PsycINFO, LILACS, Global Health Medicus and Scopus for peer-reviewed articles published between 1 January 2000 and 30 September 2023. We applied no language restrictions. We conducted a comprehensive literature search using keywords and medical subject headings terminology for tuberculosis and health literacy (Table 1). Two authors independently searched for articles in the databases. Any disagreements between the two authors were settled by a third author. To identify relevant literature not identified in the primary search, we hand-searched references of identified studies. 

**Table 1:** Search strategy to identify articles on health literacy and tuberculosis treatment adherence and outcomes

Concept	MeSH	Keywords
Tuberculosis	“Tuberculosis”[Mesh]	“Tuberculoses”[tiab] “Kochs Disease” [tiab] “Koch's Disease” [tiab] “Koch Disease” [tiab] “Mycobacterium tuberculosis Infection” [tiab] “Infection, Mycobacterium tuberculosis” [tiab] “Infections, Mycobacterium tuberculosis” [tiab] “Mycobacterium tuberculosis Infections” [tiab] “Tuberculosis”[tiab] “Tuberculosis infection*”[tiab] “Inactive TB*”[tiab] “Pulmonary Tuberculosis*”[tiab] “Koch Tuberculosis”[tiab] “Extra pulmonary Tuberculosis”[tiab] “TB”[tiab] “subclinical tuberculosis*”[tiab] “Tuberculous infection”[tiab] “Active TB”[tiab]
Health literacy	“health literacy”[MeSH Terms] OR Health literacy[Text Word]	“Literacy, Health”[tiab] “Health literacy”[tiab] “Health behaviour”[tiab] “Health education”[tiab] “Health awareness”[tiab] “Health knowledge”[tiab] “Health attitude”[tiab] “Health practice”[tiab]

MeSH: medical subject heading.

We imported all identified citations into EndNote (Clarivate, London, United Kingdom of Great Britain and Northern Ireland) and removed duplicate entries. Using Rayyan software (Rayyan, Cambridge, United States of America), two authors screened titles and abstracts of the retrieved studies to identify eligible articles. Uncertainties in eligibility were settled by a third author. Finally, two authors independently performed the full-text evaluations of the selected articles, and disagreements were resolved by a third author.

Using a self-generated standardized data extraction form, we collated relevant data and information on authors, country, study design, sample size, health literacy levels and factors determining health literacy, such as age, socioeconomic status, education and knowledge of participants.^13^ To understand the relationship between health literacy and outcomes, we sought details relevant to the three primary mediator groups delineated by the Causal Pathways Linking Health Literacy to Health Outcomes model: (i) access and use of health care; (ii) provider-patient interaction; and (iii) self-care.^13^ We categorized the outcomes reported in the studies into four types: clinical, behavioural, patient-provider communication and other outcomes. When the data were insufficient, missing or full text was unavailable, we contacted the corresponding authors of the original articles via e-mail asking them to provide the relevant information. Additionally, we extracted information for assessing the risk of bias.

### Data quality and risk of bias 

Two authors independently assessed methodological quality and risk of bias among the included studies using JBI critical appraisal tools.^14^ Potential conflicts were resolved by a third author. We then graded studies according to the scores calculated using JBI, and subsequently classified studies as low, moderate or high risk of bias. 

### Data synthesis and analysis

We summarized the extracted data using frequencies and percentages for categorical variables and median and standard deviation for continuous variables. We calculated pooled proportions of limited health literacy using Stata version no 16 (Stata, College Station, USA) and 95% confidence interval (CI) to account for variability between studies. Our analysis was weighted using a random effects model that also tested for heterogeneity and performed *I^2^* statistics. Effect sizes are expressed as odds ratio (OR) for dichotomous data. All effect estimates are drawn using a 95% CI.

## Results

**Fig. 1:**
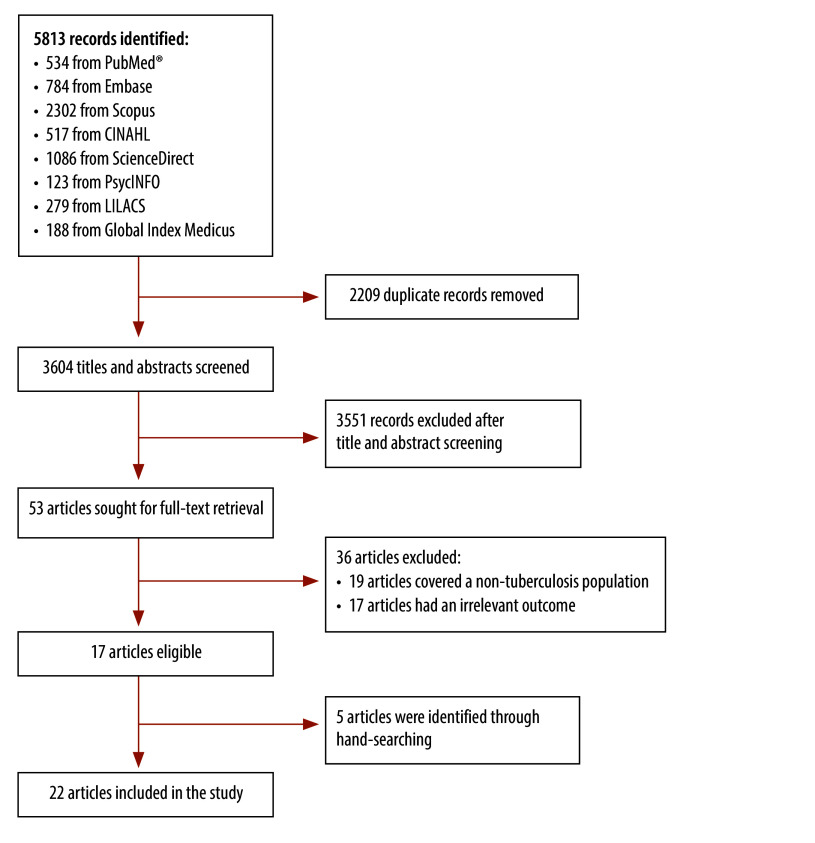
Flowchart of the selection of studies on tuberculosis and health literacy

Across all accessed databases, our search yielded a total of 5813 citations (Fig. 1). After removal of duplicates, we screened 3614 titles and abstracts and we obtained 53 publications for full-text review. Of these, 17 publications met our eligibility criteria. Because we identified five additional studies through hand-searching, the final number of included studies was 22.^9^^,^^15^^–^^35^ The most common reasons for exclusion were: studies that did not address the outcomes of interest (32.1%; 17/53) and studies that did not involve individuals with active tuberculosis (30.2%; 16/53). Two studies that addressed health literacy in health-care providers and one study in a mix of outpatient department patients were also excluded.

Nearly all the selected studies (21/22) measured as having low risk of bias (JBI critical assessment score > 70%). While one study indicated a moderate risk of bias (score 50–69%), we did not exclude any articles from our final review.

### Characteristics of studies

Most publications (17 studies) addressed health literacy and tuberculosis case detection, treatment adherence or treatment outcomes.^15^^–^^18^^,^^20^^–^^23^^,^^27^^–^^35^ Only five studies assessed links between health literacy and tuberculosis.^9^^,^^22^^,^^23^^,^^27^^,^^30^ A majority (14 studies) of the studies were observational.^9^^,^^15^^–^^18^^,^^20^^,^^21^^,^^24^^–^^26^^,^^30^^–^^32^^,^^34^ Only eight were longitudinal studies:^19^^,^^22^^,^^23^^,^^27^^–^^29^^,^^33^^,^^35^ two using data from a randomized controlled trial;^19^^,^^27^ four from a prospective cohort study;^22^^,^^23^^,^^28^^,^^35^ and two using a decentralized model for intervention.^29^^,^^33^

The included studies covered 4541 patients with active tuberculosis (range: 8–1502) across five out of the six World Health Organization (WHO) regions. The majority of research studies were from the Western Pacific Region (eight studies; 1747 patients; 38.5% of all patients);^9^^,^^22^^,^^23^^,^^28^^,^^30^^–^^32^^,^^35^ seven studies were conducted in the African Region (2144 patients; 47.2%);^16^^,^^17^^,^^20^^,^^25^^–^^27^^,^^33^ three studies each in the South-East Asia Region (369 patients; 8.1%);^19^^,^^21^^,^^24^ and the Region of the Americas (241 patients; 5.3%);^15^^,^^29^^,^^34^ and one study in the Eastern Mediterranean Region (40 patients; 0.9%).^18^

Health literacy was assessed using a variety of tools across different studies. Most studies assessed health literacy in domains such as accessing, understanding, analysing and applying health information. In addition, studies conducted in China also assessed knowledge related to tuberculosis, lifestyle and behaviour. Table 2 provides an overview of the main features of the articles.

**Table 2:** Characteristics of the included studies in the systematic review on tuberculosis health literacy

Author, year	Country	WHO Region	Study design	Sample size	Study type	Includes tools for health literacy
Cabrera et al., 2002^34^	United States	Region of the Americas	Mixed method	210	Treatment adherence	Questionnaire
Kamineni et al., 2011^21^	India	South-East Asia Region	Mixed method	219	Case detection, treatment adherence and outcome	NA
Oladunjoye et al., 2013^25^	Nigeria	African Region	Cross-sectional	74	Health literacy	Functional health literacy
Albino et al., 2014^15^	Peru	Region of the Americas	Qualitative	16	Treatment adherence	NA
Mohr et al., 2015^33^	South Africa	African Region	Randomized controlled trial	200	Treatment adherence	Questionnaire
Behzadifar et al., 2015^18^	Iran (Islamic Republic of)	Eastern Mediterranean Region	Qualitative	40	Treatment adherence	NA
Theron et al., 2015^27^	South Africa	African Region	Randomized controlled trial	1502	Treatment adherence	Questionnaire
Wilson et al., 2016^29^	United States	Region of the Americas	Cross-sectional	15	Case detection	Questionnaire
Li et al., 2016^22^	China	Western Pacific Region	Cohort	181	Treatment adherence and outcome	Chinese citizen health literacy questionnaire
Wang & Wang, 2017^28^	China	Western Pacific Region	Cohort	210	Treatment adherence and outcome	Chinese citizen health literacy questionnaire
Jie et al., 2017^23^	China	Western Pacific Region	Cohort	373	Treatment adherence and outcome	Chinese citizen health literacy questionnaire
Li et al., 2019^9^	China	Western Pacific Region	Cross-sectional	60	Health literacy	Chinese health literacy scale - tuberculosis
Asemahagn et al., 2020^16^	Ethiopia	African Region	Qualitative	21	Case detection	NA
Yang et al., 2020^30^	Republic of Korea	Western Pacific Region	Cross-sectional	206	Treatment adherence and outcome	37-item questionnaire
Qiao-Lin et al., 2020^35^	China	Western Pacific Region	Cohort	225	Treatment adherence and outcome	Health literacy management scale
Nayak et al., 2021^24^	India	South-East Asia Region	Cross-sectional	100	Health literacy	Newest vital scale
Baloyi & Manyisa, 2022^17^	South Africa	African Region	Qualitative	8	Treatment outcome	NA
Ernawati et al., 2022^19^	Indonesia	South-East Asia Region	Randomized controlled trial	50	Health literacy	HLS-EU-Q10 IDS
Kallon et al., 2022^20^	South Africa	African Region	Qualitative	29	Case detection, treatment adherence and outcome	NA
Olayemi et al., 2022^26^	Nigeria	African Region	Cross-sectional	310	Health literacy	13 item Health information literacy scale
Zhang et al., 2022^31^	China	Western Pacific Region	Cross-sectional	472	Treatment adherence	NA
Zhou et al., 2022^32^	China	Western Pacific Region	Mixed method	20	Treatment adherence	NA

NA: not applicable. WHO: World Health Organization

### Health literacy

#### Health literacy measures

In total, 14 studies measured health literacy.^9^^,^^19^^,^^22^^–^^30^^,^^33^^–^^35^ The most used measure of health literacy was the Chinese citizen health literacy questionnaire (three studies).^22^^,^^23^^,^^28^ Other measures were the Newest Vital Scale (one study),^24^ HLS-EU-ID (one study)^19^ and item-based questionnaires (nine studies).^9^^,^^25^^–^^27^^,^^29^^,^^30^^,^^33^^–^^35^ The studies differed in how investigators distinguished between health literacy levels or thresholds, either as a continuous measure or categories; that is, inadequate versus adequate or high versus low. When categorized, most of the selected studies focused on the differences between groups of the lowest and highest literacy levels. Additionally, studies differed in the types of health literacy they addressed. Most studies (85.7%; 12 studies) addressed all components (functional, interactive and critical) of health literacy.^9^^,^^19^^,^^22^^–^^24^^,^^26^^,^^28^^–^^30^^,^^33^^–^^35^ In contrast, only two studies assessed functional health literacy.^25^^,^^27^

**Fig. 2:**
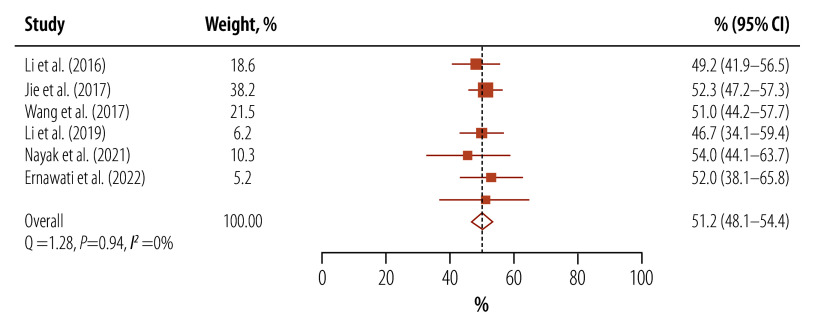
Proportion of limited health literacy among tuberculosis patients

Due to the lack of a uniform tool to assess health literacy levels across all study types, pooled mean scores were not calculated as the scoring patterns were irregular. Based on the proportion of tuberculosis patients with limited health literacy, we calculated pooled proportion of limited health literacy from eight studies as 54.9% (95% CI: 40.7–68.7).^9^^,^^19^^,^^22^^–^^25^^,^^27^^,^^28^ After excluding the two studies that assessed only functional health literacy, pooled proportion of limited health literacy was 51.2% (95% CI: 48.1–54.4; *I^2^*: 0%; Fig. 2).^9^^,^^19^^,^^22^^–^^24^^,^^28^ Two studies provided domain-wise scores for health literacy and higher scores in access to information but lower scores in understanding, analysing and applying health information.^9^^,^^30^ The reported proportion of patients with tuberculosis that had either insufficient or inadequate levels of health literacy ranged from 46.2% to 73.8%.^9^^,^^19^^,^^22^^–^^25^^,^^28^^,^^35^ Mean health literacy scores were all in the lower range, that is, all studies suggested low health literacy levels except those assessing functional health literacy. Due to low data availability, we did not perform subgroup analysis based on sociodemographic data nor groups-wise analyses.

CI: confidence interval.

Note: Limited health literacy was defined as a person's inability to understand, process and make decisions from information obtained concerning their own health.

#### Health literacy and tuberculosis

**Fig. 3:**
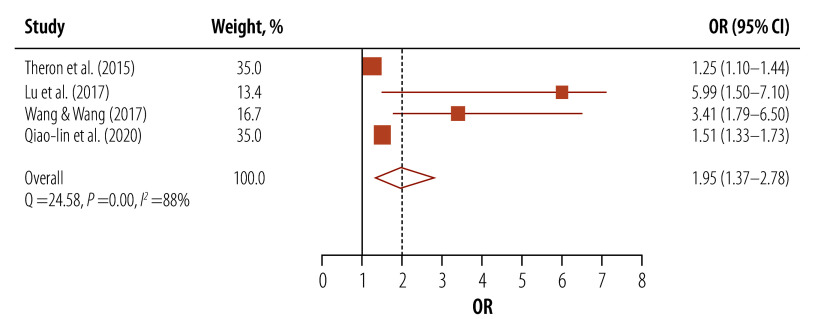
Association between health literacy and tuberculosis treatment adherence

CI: confidence interval; OR: odds ratio.

In four studies that used ORs to evaluate health literacy, a statistically significant link was found between low health literacy and suboptimal adherence to tuberculosis treatment (pooled OR: 1.95; 95% CI: 1.37–2.78; Fig. 3).^23^^,^^27^^,^^28^^,^^35^

**Fig. 4:**
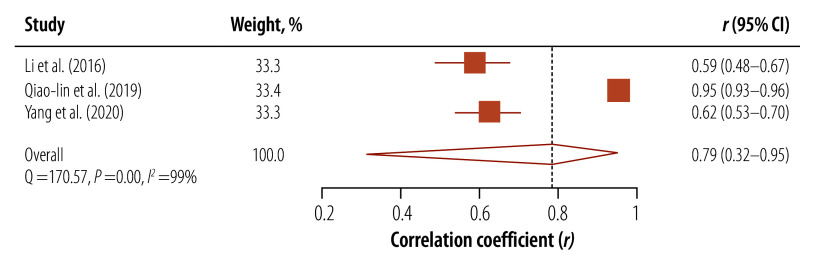
Correlation between health literacy and tuberculosis knowledge

Three studies, employing the correlation coefficient *r* to measure the relationship between health literacy and knowledge related to tuberculosis, demonstrated a statistically significant association between low health literacy and limited knowledge of tuberculosis (pooled *r*: 0.79; 95% CI: 0.32–0.95; Fig. 4).^22^^,^^25^^,^^27^ Two studies conducted in the Western Pacific Region reported a significant association between low education and limited health literacy.^22^^,^^23^ One study conducted in Republic of Korea reported a significant association between old age, low socioeconomic status and male gender with limited health literacy.^30^ Moreover, one study conducted mediation analysis and found health literacy to act as a mediator between tuberculosis knowledge and both social support and tuberculosis prognosis.^35^

CI: confidence interval.

### Outcomes related to health literacy 

Table 3 presents the health literacy outcomes reported in the studies. However, none of the studies provided data on how health literacy affects treatment outcomes such as cure rates, treatment success, default, failure, recurrence or mortality.

**Table 3:** Clinical, behavioural, patient-provider and other outcomes in studies on tuberculosis health literacy

Outcome	Mediator	No. of studies	Findings
**Clinical outcome**			
Case detection	Access and use of health care	4^15^^,^^16^^,^^18^^,^^29^	Health literacy was found to be a determinant for early diagnosis of tuberculosis and patient-initiated screening for contacts.^15^^,^^16^^,^^18^ A significant improvement was observed in case detection after intervening for the enhancement of tuberculosis literacy through video-based intervention.^29^
Treatment adherence	Access and use of health care, self-care	13^15^^,^^18^^,^^20^^–^^23^^,^^27^^,^^28^^,^^31^^–^^35^	A significant association was observed between limited health literacy and poor treatment adherence.^23^^,^^27^^,^^28^^,^^35^ This relationship was only observed in studies that adjusted for age, sex, education and treatment regimen. Intervention studies found enhancing tuberculosis literacy improved treatment adherence via educational booklets^33^^,^^34^ Six qualitative studies identified limited health literacy as a barrier to good treatment adherence.^15^^,^^18^^,^^20^^–^^22^^,^^31^^,^^32^
Treatment outcome	Access and use of health care	1^17^	One study reported limited health-care literacy contributing to non-conversion after two months of tuberculosis treatment.^17^
**Behavioural outcome**			
Tuberculosis knowledge	Patient-provider interaction, self-care	10^16^^,^^18^^,^^20^^,^^21^^,^^30^^–^^32^^,^^34^^,^^35^	Tuberculosis-related knowledge was observed to be determining health literacy among tuberculosis patients.^16^^–^^18^^,^^20^^,^^21^^,^^30^^–^^32^^,^^34^^,^^35^ Four studies adjusted for age, education, duration of tuberculosis, among others,^30^^–^^32^^,^^35^ while six did not adjust for any confounding variables.^16^^–^^18^^,^^20^^,^^21^^,^^34^
Self-efficacy	Access and use of health care, self-care	1^27^	One study reported high psychological stress influencing self-efficacy leading to poor treatment adherence.^27^
Self-care	Patient-provider interaction	2^23^^,^^28^	Two studies observed that health literacy is a determinant of effective self-care and treatment compliance among tuberculosis patients.^23^^,^^28^
**Patient-provider interaction outcome**			
Patient-provider interaction	Patient-provider interaction	2^17^^,^^20^	Two studies reported patient-provider engagement as a factor associated with health literacy.^17^^,^^20^ Provider's attitude towards tuberculosis patients and limited patient-provider engagement influenced health literacy among patients.^17^^,^^20^
**Other outcome**			
Understanding of health information	Patient-provider interaction	9^15^^–^^18^^,^^20^^,^^21^^,^^31^^,^^32^^,^^34^	In nine qualitative studies, failure to comprehend health information was reported as a major factor influencing health literacy level.^15^^–^^18^^,^^20^^,^^21^^,^^31^^,^^32^^,^^34^ Three studies reported factors such as inconsistent messages from health-care providers, language barriers, medical jargon and the use of technical language, as major barriers to health literacy.^15^^–^^17^
Provider skill level	Access and use of health care, self-care	3^15^^,^^16^^,^^18^	Three studies observed that health literacy is influenced by limited knowledge and skills among health-care providers^15^^,^^16^^,^^18^
Organizational factors	Patient-provider interaction	2^16^^,^^18^	Two studies reported various organizational factors influencing health literacy such as a lack of resources, limited space for assessing patients, limited operating time of health centres and shortage of health-care workers.^16^^,^^18^

#### Health literacy interventions

Among the selected studies, only one study assessed the role of an intervention to improve health literacy for patients with tuberculosis.^19^ The research, conducted in Indonesia, documented a positive impact of an educational booklet on the health literacy levels of tuberculosis patients. However, the authors did not specify which tool was used to assess health literacy nor which areas of health literacy were improved by the intervention.

## Discussion

Our systematic review provides initial broad estimates of health literacy levels in patients with active tuberculosis. Our evidence predominantly applies to countries in the African, South-East Asia and Western Pacific Regions that also bear a substantial burden of tuberculosis. We identified a significant association between health literacy and adherence to tuberculosis treatment. Factors such as knowledge of tuberculosis, self-care practices, self-efficacy, patient-provider engagement, understanding of health information and the abilities of providers were found to influence health literacy.

We observed that half of tuberculosis patients had limited health literacy. Limited health literacy has also been observed among patients diagnosed with human immunodeficiency virus (HIV) infection and diabetes (31.4% and 28.3%, respectively).^36^^,^^37^ Interestingly, a bi-directional relationship exists between HIV infection and tuberculosis, as well as diabetes and tuberculosis.^38^ Current literature suggests health literacy interventions can potentiate improvements in knowledge, behaviour skills and self-management practices for people living with HIV and diabetes.^39^^,^^40^

In light of the evidence documenting limited health literacy among co-morbid tuberculosis patients, concrete health literacy interventions are required.^7^ Further, improving health literacy could be an effective way to prevent co-morbidities in individuals with chronic disease, suggesting improved health literacy could reduce acquisition of co-morbid diseases commonly associated with tuberculosis such as diabetes and HIV, among others.^41^ Thus, health literacy is beneficial in any chronic disease scenario where patient empowerment, active involvement in self-management, self-care, timely care seeking, patient navigation and patient engagement, are essential.^2^

We observed that individuals with active tuberculosis and limited health literacy had 1.5 times higher odds of poor treatment adherence compared to those with adequate health literacy. Individuals with limited health literacy may not comprehend the importance of completing the entire treatment regimen and the risk of acquiring drug resistance.^4^ Hence, poor treatment adherence contributes to unfavourable treatment outcomes including failure, relapse, recurrence or death.^42^ In India, non-adherent tuberculosis patients had an estimated four times greater likelihood of unfavourable treatment outcomes (OR: 4.0; 95% CI: 2.1–7.6).^43^ A meta-analysis of clinical trials revealed that missing more than 10% of doses is associated with a sixfold increased risk of unfavourable tuberculosis outcomes.^44^ Nonadherence has also been reported as a risk factor for drug-resistant tuberculosis.^7^^,^^45^ Furthermore, reducing nonadherence could have a larger epidemiological impact on tuberculosis incidence in high-burden countries than in low-burden countries.^46^

Health literacy empowers patients to adhere to the treatment plan, accelerating the chances of good treatment outcomes.^4^ Additionally, various patient-centric factors influence tuberculosis treatment adherence. Knowledge related to tuberculosis and social support are key determinants of treatment adherence among tuberculosis patients.^47^ In Ethiopia, patients with inadequate knowledge had an estimated 4.11 (95% CI: 1.57–10.75) times greater risk of poor treatment adherence.^48^ Our review suggests that individuals with a strong understanding of tuberculosis generally exhibit better health literacy than those with less knowledge. Additionally, our findings indicate health literacy may serve as a link between tuberculosis knowledge and social support.

Another patient-centric factor for poor treatment adherence and timely diagnosis is delayed access to care due to stigma.^10^ Stigma associated with tuberculosis, often stemming from various myths and misinformation, may be mitigated by health literacy. Improved understanding can reduce both implicit and explicit stigma, encouraging individuals to promptly seek care.^10^ Thus, health literacy emerges as a crucial factor linked to both treatment adherence and tuberculosis-related knowledge. One study showed that health literacy also mediates the provision of social support,^35^ such as financial aid, nutritional advice and medication assistance, all of which can influence treatment outcomes.

We observed that high levels of self-care in tuberculosis patients was determined by knowledge and the ability to understand health information. Self-care depends on the ability of the health-care systems and providers to teach, as well as the patients to learn effective self-management skills.^15^ Our observations indicate that tuberculosis patients often struggle with understanding health-care information, impeding their ability to effectively manage the condition.

Other researchers have noted that tuberculosis knowledge, health education and family support are positively correlated with high levels of self-management.^49^ Self-management of tuberculosis patients seems to be a critical patient-centric strategy for enhancing treatment adherence and outcomes.^49^ We also observed that those aged 60 years and older and low socioeconomic status have lower health literacy levels, which could affect decision-making, self-management and treatment adherence. Socioeconomic status does not directly affect health; however, health literacy acts as a mediator between socioeconomic status and health, quality of life, health outcomes and the use of preventive services.^5^

Thus, as health literacy is a modifiable risk factor, enhancing health literacy can improve equity in health care. Our findings also show that subpar patient engagement and insufficient skills among health-care providers have an impact on the health literacy of tuberculosis patients. Health literacy intertwines with a patient’s education level, intelligence, and communication abilities, alongside a provider’s capability to use language and examples that are culturally, relationally and situationally appropriate for patient comprehension.^1^ Therefore, the development of targeted health literacy interventions is essential. These should be multifaceted, aiming to empower patients, increase their level of engagement in health-care decisions, and improve the quality of physician-patient communication. Additionally, ensuring that such interventions are evaluated across various health-care environments tailored to tuberculosis management is critical. Observations from international case studies, such as the improvements in patient outcomes for hepatitis C in Egypt due to improved health literacy, suggest that analogous strategic investments in tuberculosis-related health literacy could yield comparably positive results.^50^

Our review has some limitations. First, due to the lack of a uniform tool to assess the health literacy level, we could not pool health literacy scores, including domain-wise scores, to obtain extant levels of health literacy among tuberculosis patients. Second, as many studies did not report on the outcomes of tuberculosis, we could not assess the impact of health literacy on tuberculosis outcomes such as cure rate, failure, recurrence or mortality. Third, the variety of health literacy tools measuring different aspects of health literacy restricted the extensiveness of the meta-analysis. We could not perform a subgroup meta-analysis based on demography, co-morbidity nor drug resistance. Finally, given the limited number of studies included in the analysis of health literacy and tuberculosis, there is a potential for heterogeneity within the findings.

This review highlights that health literacy is associated with treatment adherence and disease understanding. Health literacy-focused interventions tailored to different contexts are needed to foster patient empowerment and improve health outcomes. We suggest that tuberculosis interventions should extend beyond knowledge enhancement to include skills-building and health information comprehension. Future research should explore health literacy’s effects on co-morbidities and other tuberculosis-related issues. Moreover, a harmonized approach to measuring health literacy is essential. Improved health literacy supports individuals in taking charge of their health, and aids providers in delivering inclusive care. Literacy also stands to lessen health inequities and bolster public health. Well-informed tuberculosis patients can contribute significantly to elimination efforts, benefiting the wider community. 
